# Forging a path to mesoscopic imaging success with ultra-high field functional magnetic resonance imaging

**DOI:** 10.1098/rstb.2020.0040

**Published:** 2020-11-16

**Authors:** Kimberly B. Weldon, Cheryl A. Olman

**Affiliations:** 1 Department of Psychiatry and Behavioral Sciences, University of Minnesota, Minneapolis, MN 55455, USA; 2 Center for Magnetic Resonance Imaging, University of Minnesota, Minneapolis, MN 55455, USA; 3 Department of Psychology, University of Minnesota, Minneapolis, MN 55455, USA

**Keywords:** ultra-high field, fMRI, layer dependent, cortical columns, spatial specificity

## Abstract

Functional magnetic resonance imaging (fMRI) studies with ultra-high field (UHF, 7+ Tesla) technology enable the acquisition of high-resolution images. In this work, we discuss recent achievements in UHF fMRI at the mesoscopic scale, on the order of cortical columns and layers, and examine approaches to addressing common challenges. As researchers push to smaller and smaller voxel sizes, acquisition and analysis decisions have greater potential to degrade spatial accuracy, and UHF fMRI data must be carefully interpreted. We consider the impact of acquisition decisions on the spatial specificity of the MR signal with a representative dataset with 0.8 mm isotropic resolution. We illustrate the trade-offs in contrast with noise ratio and spatial specificity of different acquisition techniques and show that acquisition blurring can increase the effective voxel size by as much as 50% in some dimensions. We further describe how different sources of degradations to spatial resolution in functional data may be characterized. Finally, we emphasize that progress in UHF fMRI depends not only on scientific discovery and technical advancement, but also on informal discussions and documentation of challenges researchers face and overcome in pursuit of their goals.

This article is part of the theme issue ‘Key relationships between non-invasive functional neuroimaging and the underlying neuronal activity’.

## 1. Introduction

Functional magnetic resonance imaging (fMRI) has been a prolific tool for cognitive and neuroscientific research since its introduction in the early 1990s [1–3]. Imaging systems at 3 Tesla (3T) have become standard in both clinical and research applications, and, in pursuit of high-resolution imaging facilitated by ultra-high field (UHF) strengths, dozens of 7 Tesla (7T) systems have been installed globally [4]. As the technology has improved by way of increasing field strength-dependent signal-to-noise ratio (SNR) [5–7], functional contrast-to-noise ratio (CNR) [8,9] and spatial specificity (for review see [10]), more details of the functional architecture of the brain are available for study. Indeed, several studies have found that current fMRI technology allows for studying the functional organization of cortex at a mesoscopic (sub-millimetre) scale, which can reveal cortical columns or layers (see the special issue [4] and [11,12] for recent reviews).

Despite these advances, fundamental challenges of fMRI remain. Spatial specificity is considered a strength of fMRI as a non-invasive neuroscientific technique compared to magnetoencephalography (MEG) or transcranial magnetic stimulation (TMS). That said, the BOLD signal is an indirect measure of neural activity that relies on blood oxygenation, and high-resolution imaging requires careful consideration of how the brain's micro- and macrovasculature contribute to the spatial characteristics of the signal. Additionally, using sub-millimetre image resolution to make inferences about where signal occurs requires high precision in localizing the function with regard to anatomical landmarks (e.g. grey matter (GM) and white matter (WM) boundaries, pial surface of the GM). In this paper, we illustrate the mesoscopic imaging capabilities of UHF fMRI, address current pitfalls and emphasize how different acquisition methods affect spatial specificity in fMRI data.

## 2. Achievements and limitations of sub-millimetre resolution

### (a) Separation of neural subpopulations by columnar organization

**Figure 1.**
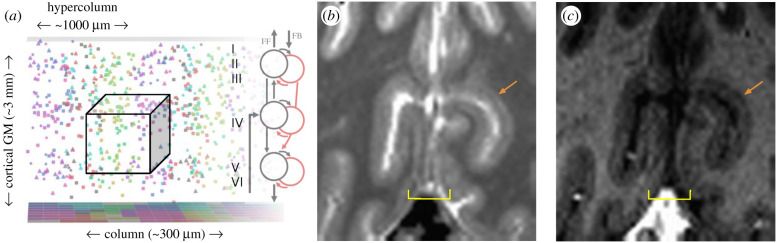
Spatial scales of interest. (*a*) Simulation of a hypercolumn in cortical grey matter (GM). Neurons with similar response properties are indicated by colour and clustered in columns 200–500 microns in diameter, spanning the cortical depth. Within a column, at the microscopic level, there is striking diversity in response properties and cell types. Here, circles indicate inhibitory neurons, squares indicate excitatory neurons with only local projections, and triangles indicate excitatory neurons with long-range projections. Microscopic resolution would be required to separate cell types, but mesoscopic resolution, the target of many current UHF fMRI experiments, can separate columns and layers. (*b*) Resolving stria of Gennari at 7T. T_1_ maps from a 10-min MP2RAGE acquisition at 7T shows clear hypointensities (orange arrow) in the calcarine sulcus, which presumably arise from layer IV. Importantly, the dark stripe is not evident everywhere. (*c*) A T_1_-weighted acquisition with parameters optimized for contrasting GM and WM at 7T [15] also shows the layer IV stripe (orange arrow), but, again, it is not always clearly visible. Yellow brackets indicate regions near the midline that are particularly susceptible to banding artefacts caused by the sagittal sinus, which look similar to the stria of Gennari. These images demonstrate that, while imaging of the stria is feasible, the results are sometimes ambiguous and location and orientation dependent. (Online version in colour.)

A key advantage of UHF fMRI is the ability to increase the spatial resolution at which data are acquired. At conventional field strengths, a typical voxel size in human fMRI of 2–4 mm per dimension is used for investigations into cortical activation at the macroscopic level (i.e. between brain regions). Neuron density in cortical GM is roughly 50 000 neurons mm^−3^ [13,14], and microscopic resolution (i.e. at the single-cell level) is currently beyond the reach of *in vivo* fMRI experiments. The improving technology of UHF fMRI, however, facilitates high-resolution imaging with voxel dimensions smaller than a millimetre, which opens the door for detecting responses at a mesoscopic level, from neuronal subpopulations smaller than was previously possible, such as columns or layers (figure 1).

In many regions of cortex, neurons with similar response properties are clustered in vertical columns 200–500 microns in diameter, spanning the six histological layers of cortical depth. These cortical columns have been considered a fundamental element of cytoarchitecture since their discovery [16]. Until recently, most of the knowledge about the structure and function of cortical columns has come from animal models using invasive or anatomical techniques. Early demonstrations of ocular dominance columns (ODCs) in humans came from post-mortem cytochrome oxidase staining of brains from patients who had become blind in one eye before their death [17]. The high-resolution capabilities of UHF MRI and the resulting smaller voxel sizes make it possible to study these structures in the human brain *in vivo*. Columns for ocular dominance [18–20], orientation [21] and temporal frequency [22] have been visualized in human V1. Colour-selective columns in V2 and V3 [23] and columns for motion in human middle temporal area (MT) [24] have also been visualized. Columnar organization of sound frequency processing in the human auditory cortex has been demonstrated using sub-millimetre acquisitions [25].

These important works demonstrate the potential of UHF fMRI as a tool in cognitive neuroscience research. However, it is important to note that the functional purpose for columnar organization is not fully understood ([26], but see [27]). For example, ODCs appear in some species but not others, and even within species there is variability in the expression of ODCs without demonstrable differences in visual function [28]. Additionally, the expression of cortical columns is difficult to verify *in vivo* without acquiring functional data across multiple days and/or scanning sessions to confirm reproducibility.

### (b) Separation of neural subpopulations by laminar organization

Voxel sizes smaller than one millimetre also allow for the examination of relative contributions of deep, middle or superficial layers of the GM of neocortex. The six layers of the GM ribbon are bounded by the pial surface and the WM boundary. Neurons are distributed through the GM depth according to connectivity patterns. We know, for instance, from single-cell and anatomical studies of macaque V1 that feed-forward connections terminate primarily in layer IV [29] and feedback connections from higher visual areas terminate primarily in superficial and deep layers, while avoiding layer IV [30,31]. Typical sub-millimetre resolution of 0.8 mm is not sufficient to resolve the individual histological layers as the total thickness of the GM ribbon itself is between 1.4 and 4.5 mm [32], but the presence of objective anatomical boundaries (i.e. the GM/WM boundary and the pial surface) means that layer ‘location’ can be approximated through calculated measures with more confidence than cortical columns.

Two possible ‘solutions’ for estimating layer depth are equidistant (e.g. see [33,34]) or equivolume [35] calculations. In equidistant solutions, depth location for each lamina in GM is calculated keeping the relative depth constant between the outer boundaries. This approach assumes the thickness of laminae remains constant irrespective of cortical folding. However, in folded cortex layer thickness is not constant, but changes such that laminae volume is preserved ([36] as cited in [35]). An equivolume approach compensates for the variable thickness of each lamina and preserves their volumes along the GM ribbon. In addition to the WM and pial surface bounding the GM, the highly myelinated stria of Gennari [37] can be resolved *in vivo* (figure 1*b* [38]) and nicely aligns with the middle of the equivolume solution [39].

The presence of objectively identifiable landmarks and the functional significance of the GM have led to layer-dependent fMRI becoming an enormously popular subfield in UFH fMRI. Several studies have taken advantage of UFH fMRI capabilities to resolve functional responses at different cortical depths [33,39–47]. Visual stimuli evoke patterns of BOLD response across cortical depth in V1 [39,43] and MT [40]. Further, different stimulus conditions evoke variations in the distribution of the BOLD response across cortical depth for visual stimuli [33]. Depth profiles have also been demonstrated in studies investigating somatosensory stimuli [41], working memory [45], language processing [46] and feedback responses in early visual cortex [33,42,47].

### (c) Information representation without explicit separation of neural subpopulations

Because even the smallest voxel contains a diverse population of neurons, analysing the information contained in voxel populations as opposed to average response amplitudes has become an increasingly popular strategy for maximizing fMRI sensitivity to fine-grained cortical activity. Human neuroimaging studies show reliable orientation information can be decoded in V1 at conventional (3 mm) resolution [48,49]. There is growing interest in using the high resolution afforded by UHF to improve upon the ability of multivariate pattern analysis (MVPA) methods to extract reliable signals from distributed patterns of brain activity [48–50]. Support vector machine learning approaches appear to be promising in UHF fMRI. For example, Bergmann *et al*. [51] used MVPA of high-resolution (0.8 mm isotropic) fMRI data from human participants to show differences in laminar activity for mental imagery and illusory percepts. They found that low-level feedback in V1 during illusory perception occurred more in superficial layers, compared to mental imagery, which occurred more in deep layers. In another preliminary study, three different classifiers were tested on data acquired at 0.8 isotropic resolution and on artificially misaligned data to test the robustness of the classification accuracy [52]. A one-voxel shift of the test region of interest (ROI) from the training ROI led to a significant decrease in decoding accuracy, suggesting multivariate decoders can be as precise as the nominal resolution of single voxels (here, 0.8 mm isotropic), unlike problems associated with typical analyses [52]. Although, as detailed below, there are still many questions to be answered about the true spatial resolution of UHF fMRI data, these studies show that high-resolution data give us access to discoveries at the mesoscopic level that would be beyond reach if acquired at conventional resolutions.

### (d) Challenges for accurate localization of mesoscopic functional magnetic resonance imaging signal

One inherent pitfall of UHF MRI is an increase in inhomogeneities in the static magnetic field (B_0_) that comes with higher field strength [53]. Long read-out times often used in UHF result in image distortion anywhere the field is perturbed by air, bone or a large vein or sinus owing to phase errors accumulated during the read-out time. FMRI data are most commonly acquired with echo-planar imaging (EPI), which are particularly sensitive to B_0_ inhomogeneties. The long image read-out time results in geometric distortions in the images themselves, especially in the phase encode the direction of the EPI read-out [53,54]. This is particularly problematic for depth-resolved fMRI because, as previously discussed, cortical layers must be approximated between pial and WM boundaries. Because the contrast between GM and WM is relatively poor in EPI images, these boundaries are often defined in anatomical volumes acquired with different pulse sequence (usually MP2RAGE [55], or MP-RAGE [56]) with different distortions e.g. [34,57]. When anatomical images are acquired separately from the functional data, researchers face the additional challenge of optimizing registration between different datasets (i.e. cross-modal registration).

There are several algorithms for registering functional and anatomical images implemented in freely available packages used for analysing fMRI data (e.g. AFNI [58], FSL [59], SPM (https://www.fil.ion.ucl.ac.uk/spm), FreeSurfer [60]). For example, the local Pearson correlation cost function implemented in AFNI is based on maximizing negative correlations between functional and anatomical data [61]. Boundary-based registration algorithms implemented in FSL and FreeSurfer have shown promise at aligning whole-brain images to images with limited coverage [62]. More recently, a recursive application of boundary-based registration has shown promise in automating nonlinear distortion correction [63]. AFNI's *3dQwarp* function is another nonlinear registration tool used in distortion correction, although visual inspection remains a necessary step for all registration attempts. A systematic comparison of registration algorithms is beyond the scope of this paper, although in our hands, the boundary-based registration algorithm implemented in FSL (flirt -cost bbr) and local Pearson correlation implemented in AFNI (3dAllineate -c lpc) have proven more reliable than other approaches [64].

Many of the challenges inherent to cross-modal registration can be alleviated with the acquisition of anatomical volumes in the same scanning session as the functional data. This practice reduces the need to correct for gradient nonlinearities [65]. The MP2RAGE sequence results in segmentable T_1_-weighted anatomical images with reduced sensitivity to inhomogeneities in radio frequency transmit profiles (i.e. B_1_ inhomogeneities [55]). That said, cortical reconstruction efforts of anatomical data acquired at UHF may be less reliable for those studies using a surface coil with a limited field of view. Defining surfaces and GM boundaries from anatomical images acquired during a separate scanning session may be the most practical approach, though distortion in the functional images remains a problem.

One compelling method to resolving the cross-modal registration challenges is the acquisition of T_1_-weighted volumes that have the same read-out (i.e. same image distortions) as the functional data [66,67] in the same scanning session. In a best-case scenario, the definition of GM boundaries can be defined in those images distortion-matched to the functional data but with improved GM/WM contrast. Where direct segmentation of the data cannot be done accurately (e.g. owing to the use of a surface coil), the T_1_-weighted functional data can be used to guide and optimize cross-modal registration of anatomical reference volumes acquired in separate scanning sessions. Improved contrast in the T_1_-weighted EPI volumes relative to typical functional data leads to more accurate registrations to anatomical reference. The registration matrix resulting from this initial registration can be used to guide the registration of anatomical reference volumes to UHF functional data, ultimately resulting in more accurate localization of functional voxels with respect to the GM ribbon.

Even after optimizing the definition of GM boundaries, making accurate inferences of the neuronal response at an assigned depth remains a significant challenge of laminar analysis of fMRI data. Because the BOLD signal is reflective of the haemodynamic response to the neural activity rather than a direct measure, the BOLD signal is inherently dependent on the underlying vasculature. Gradient echo (GE) EPI remains the most popular pulse sequence type in UHF fMRI owing to its high CNR compared to T_2_-, cerebral blood flow (CBF) and cerebral blood volume (CBV)-weighted techniques, and its whole-brain coverage capabilities compared to three-dimensional gradient-and-spin-echo (GRASE) (see later sections for an in-depth discussion). However, the T_2_*-weighted GE signal is predominantly generated from venous microvasculature and macrovasculature [68,69]. Ascending veins carry deoxygenated blood towards the pial surface and pial veins. Thus, the anatomical distribution of ascending and pial veins coupled with how laminar profiles are ‘read’ (i.e. per cent signal change differences at relative depths between WM boundary and pial surface) result in a GE signal biased towards more superficial layers. In essence, a signal that we expect to find in middle layers has the potential to be expressed as activation in middle and superficial layers (for reviews see [70,71]). It is important to carefully consider the impact of acquisition decisions on the expression and spatial specificity of the MR signal.

## 3. Optimizing spatial specificity in ultra-high field functional magnetic resonance imaging

**Figure 2.**
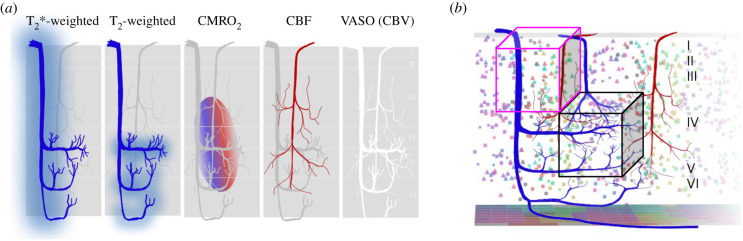
Vascular structure and functional MRI contrast mechanisms. (*a*) While T_2_*-weighted approaches (e.g. GE EPI) have strong contributions from macrovasculature, other functional MRI technologies are biased toward the microvascular signal, which is regulated at a scale appropriate for capturing the mesoscopic neuronal structure. Blue lines represent veins; red lines represent arteries. Acquisition techniques weighted toward the microvascular signal often have relatively low CNR and therefore require long acquisition times or high field strength to be successful. VASO, vascular space occupancy. (*b*) Many studies that use high-CNR T_2_*w fMRI rely on the fact that voxels can be separated into low-resolution voxels that sample large veins (pink cube) and high-resolution voxels that are dominated by microvascular signal (black cube). (Online version in colour.)

The vast majority of fMRI techniques measure changes in cerebral metabolic rate of oxygenation (CMRO_2_), CBF and/or CBV (figure 2). Thus, the field has given a great deal of attention to the structure of brain vasculature and how both intra-vascular and extra-vascular effects create the BOLD signal [72–74]. For high spatial specificity, it is essential to minimize the contributions from pial vessels and large venuoles, targeting instead the blood flow, volume and oxygenation changes in the small arterioles, capillaries and venuoles of the microvasculature. This is, of course, more easily said than done. We briefly review the advantages and challenges of common acquisition techniques in UHF fMRI.

### (a) Two-dimensional spin-echo echo-planar imaging

It has been convincingly argued that, at least in theory, T_2_-weighted techniques are superior to T_2_*-weighted techniques because large-vein extra-vascular signals are eliminated by a refocusing pulse during acquisition [20,75,76], and intra-vascular signals produce very small contributions to the signal because of short T_2_ at 7 T [73,77]. We have had good success in the past with simple two-dimensional spin-echo (SE) EPI acquisitions, using them both at a moderate resolution to demonstrate the advantage of removing large-vein signal in macroscopic mapping application [78] and at sub-millimetre resolution to map the columnar structure of visual cortex in a participant who lacked an optic chiasm [79].

SE EPI has definite advantages over GE, but it has not dominated the field because of several limitations. First, SE, like GRASE and vascular space occupancy (VASO), uses an additional 180° refocusing radio frequency (RF) pulses to eliminate the large-vein signal [20,80]. As a result, when a whole-head RF coil is used for data acquisition, one quickly runs up against specific absorption rate (SAR) limits (set independently by the scanning system) that determine how much power can be delivered safely by the pulse sequence. This is not an insurmountable problem, but it is a consideration. The restrictions that RF power deposition places on coverage can be somewhat ameliorated by using smaller transmit coils, which use less power because they do not try to deliver power to the whole head. Surface transmit coils are an excellent choice for sensory and motor studies, although naturally inappropriate for studies desiring whole-brain coverage. In Olman *et al*. [79], acquiring 18 slices per second using a surface coil yielded a 50% increase in coverage compared to similar acquisitions using a volume coil. This approach provided adequate coverage for V1. However, since the contrast in SE EPI acquisitions requires well-calibrated RF pulses and the flip angle varies significantly throughout the cortex—especially at UHF and with surface coils—only a portion of the brain volume covered by the pulse sequence (roughly 50%) provided adequate CNR for analysis. This limitation is not a problem for applications focused on a single visual area and well-placed slices with a well-calibrated coil can provide beautiful images in which the fMRI contrast is dominated by small veins.

### (b) Three-dimensional GRASE

**Figure 3.**
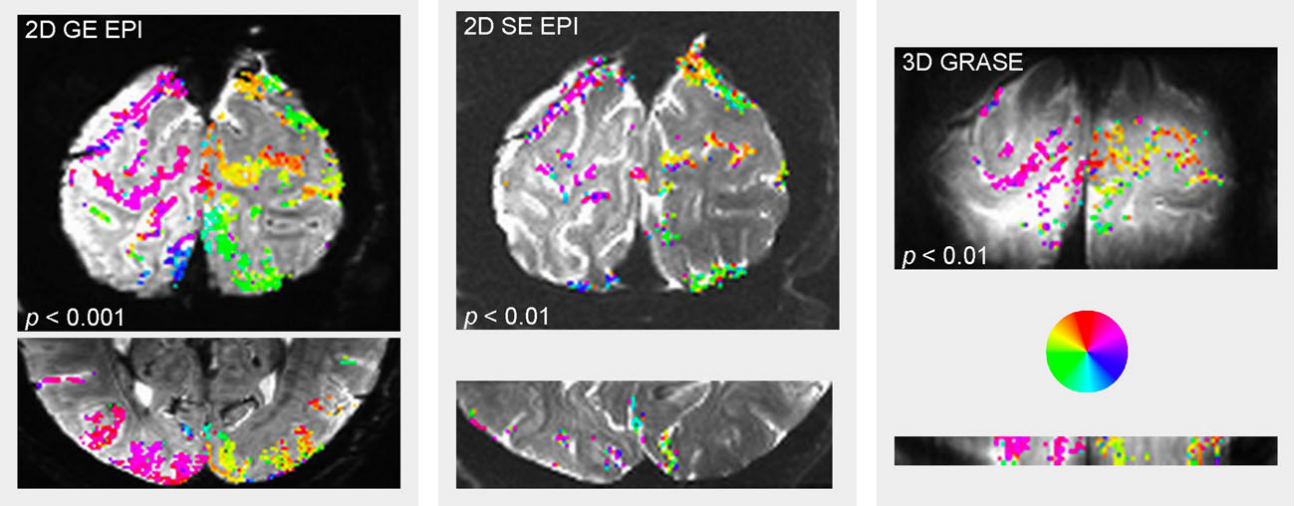
Responses during a population receptive field (pRF) mapping experiment in two-dimensional GE EPI, two-dimensional SE EPI and three-dimensional GRASE. All three acquisitions had 0.8 mm (isotropic, nominal) resolution. Each pRF mapping scan contained 16 sweeps of bars with dynamic content, each sweep lasted 16 s, with 4 s rest between, for a total scan duration of 324 s. The two-dimensional GE EPI and three-dimensional GRASE data were acquired with a 2 s TR and there were 4 pRF scans in the scanning session; the two-dimensional SE data were acquired with a 3 s TR, and there were 6 pRF scans in the scanning session, so all results show analysis of 648 TRs. F-statistics representing response variance explained a visual encoding model fit to the data (using standard routines provided by AFNI) were used to threshold the colour overlay at the single-voxel *p*-value indicated for each modality; subsequent cluster-wise correction controlled for multiple comparisons. Colour overlay indicates the estimated polar angle coordinate of the region of the visual field represented by the neural population in each voxel. All modalities agree on the retinotopic position of the population receptive field in each voxel. GE EPI shows the greatest sensitivity, followed by three-dimensional GRASE and two-dimensional SE. For details see electronic supplementary material. (Online version in colour.)

The advantage of T_2_-weighted three-dimensional GRASE is that it uses an inner volume excitation technique: the excitation pulse and 180° refocusing pulses are acquired on orthogonal dimensions to define a three-dimensional slab in which signal is acquired. Signal from outside that slab decays during the long read-out time, during which time signal inside the slab is kept alive by a train of refocusing pulses; additional crusher gradients ensure that signal from outside the imaging volume does not contaminate the ROI. This technique makes it possible to target a particular region and spend valuable acquisition time acquiring only relevant data. The small field of view also equates to short read-out times. The short read-out time of three-dimensional GRASE reduces its vulnerability to distortion (in the data shown in figure 3, the single-slice read-out time for the GRASE images was 22 ms while the read-out time for the GE and SE data was approximately 40 ms). The CNR and spatial specificity of three-dimensional GRASE are also potentially enhanced by stimulated echoes [81] and removal of macrovascular signal by crusher gradients, as described in detail elsewhere [82].

Three-dimensional GRASE does have disadvantages. Data from the edges of the slab cannot be analysed owing to signal drop-off from the excitation pulse profile in the phase-encode direction (note the soft edges on GRASE images in figure 3), aliasing in the slice direction, and/or signal drop-off owing to the refocusing pulse profiles. Three-dimensional GRASE acquisitions also necessitate a limited field of view. While several studies have demonstrated moderate success with single-slab three-dimensional GRASE applications [33,83,84], adequate coverage for fMRI applications requires multi-slab acquisitions. In principle, this is feasible. In practice, however, stitching together slabs with soft edges, and/or registering them to reference anatomical images, is quite a challenge. The goals of a given experiment will inform the experimenter whether these challenges are worth it, and three-dimensional GRASE offers a low-distortion, high contrast, small-vein biased method of doing fMRI.

### (c) Vascular space occupancy

Measurements other than the BOLD contrast, such as CBF and CMRO_2,_ have also been in development. CBV measurements using the VASO [85] technique have been used successfully in laminar fMRI in humans (for recent discussions see [86,87]). The VASO technique uses an inversion recovery pulse sequence to null blood signal while maintaining part of the tissue signal. Like other alternatives to GE-BOLD, VASO has lower CNR and temporal resolution, which leads to limitations on brain coverage. It is also the case that the fMRI contrast in VASO can be a mixture of T_2_*- and T_1_-weighted effects, which if not handled carefully will degrade the signal (see [88] for discussion) or complicate interpretation. However, T_2_* contamination can be subtracted out of the images (at the expense of temporal resolution [89]), and some of the most convincing demonstrations of depth-resolved fMRI have been performed with VASO. The discussion about how the signal should be fully characterized is on-going [90] but the elegance of the laminar signal is self-speaking [91], as are the neuroscientifically convincing ways in which the depth-dependent signal is modulated according to task demands [92].

### (d) Gradient echo is hard to leave behind

**Figure 4.**
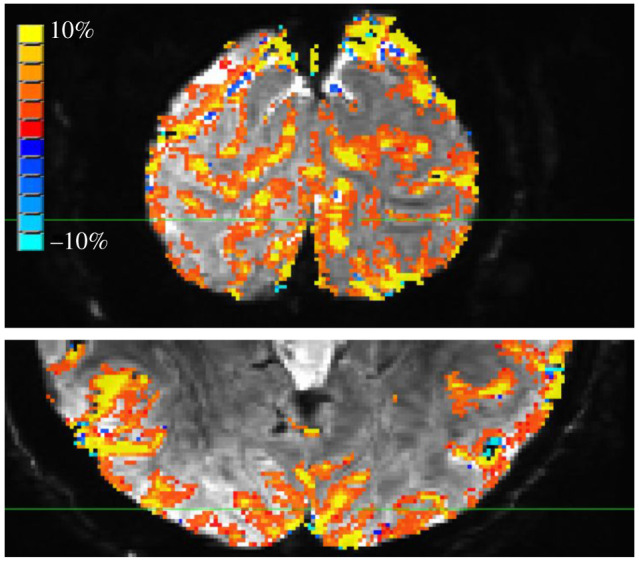
Robust activation in T_2_*w acquisition during free viewing of a movie is restricted to the GM ribbon but shows evidence of dominance by large veins. The participant watched the movie in 12 s blocks, with 12 s rest in between, for a total of 16 min. Colour indicates voxels significantly modulated by visual stimuli (*p* < 0.001 for individual voxels; corrected for multiple comparisons by requiring clusters larger than 20 voxels, for a cluster-wise false-positive rate of 0.001). (Online version in colour.)

Despite its criticisms, GE EPI has remained the fMRI workhorse, even for studies that seek the sub-millimetre resolution necessary to resolve depth-dependent fMRI signals [43,44,93,94]. The cause is twofold. First, GE is relatively easy to implement and acquire. Second, for a given amount of time in the scanner, GE acquisitions yield higher CNR compared to SE or GRASE acquisitions (figures 3 and 4) (for details on the GE acquisition, see [64]). The perpetual argument against T_2_*-weighted techniques, however, is that much of the robust CNR (and thus, resulting inference about neuronal responses) is biased by signal from large veins (e.g. see the punctate yellow regions in figure 4). In pursuit of optimizing the functional specificity of GE's high CNR, much attention has been given to different methods for addressing the ‘large vein problem’ in T_2_*-weighted images.

### (e) Addressing the ‘large vein’ problem in T_2_*-weighted acquisitions

Voxels where the largest veins dominate the BOLD contrast also have high noise levels relative to the baseline image intensity; thus, these voxels can be identified and removed. Voxels can be identified by masks based on image SNR [95], BOLD CNR [34,66] or both [84] and removed from analysis. Voxels with extraordinary blurring owing to either large veins or proximity to strong local field perturbations can also be identified by their functional response properties. For example, Muckli *et al*. [44] identified and removed vein-contaminated voxels by masking out voxels with unusually large receptive fields.

Instead of, or in addition to, masking out regions of the image where the large-vein signal dominates, there are also now some established methods for modelling the vein signal based on an understanding of its characteristics, thus using a principled approach to account for large-vein contributions during analysis. In an event-related dataset, Kay *et al*. [94] took the approach of modelling data with regressors that had both short and long latencies, showing that the variance explained by short-latency regressors had better spatial specificity. For laminar applications, a series of publications provided convincing demonstrations that penetrating intracortical venuoles create a directional blurring of the fMRI signal through the cortical depth, such that responses measured in superficial voxels are actually the sum of deep and superficial responses [96,97]. Deep responses, on the other hand, are more likely to be uncontaminated (although there are large veins running parallel to the GM/WM boundary whose contributions to the depth-dependent fMRI response have yet to be quantified [98]). This is a logical consequence of the vascular structure [99], and a key insight to bear in mind when looking at all published laminar profiles reliant on the BOLD signal, regardless of the acquisition approach.

## 4. Acquisition considerations

One lesson demonstrated in a recent dataset was the importance of paying attention to all acquisition details, not just the obvious ones (e.g. nominal resolution, coverage and the calibration of flip angle across the field of view). When setting up a new protocol, it takes some work to find an acceptable balance between spatial and temporal resolution. In a two-dimensional sequence, increasing the number of slices decreases the temporal resolution; in a three-dimensional sequence, increasing the slab thickness increases the SNR (owing to an increase in the number of samples contributing to the inverse Fourier transform that creates the image) but decreases the temporal resolution. Increasing in-plane resolution increases read-out time. For a T_2_*-weighted acquisition, the goal is to match T_2_* in the tissue in order to optimize BOLD contrast, but T_2_* in GM at 7 T is approximately 25–35 ms [5,100,101]. Reading out a matrix large enough to provide sub-millimetre resolution in less than 40 or 50 ms requires strong gradients. Strong gradients generate strong acoustic noise, and the wise experimenter will use a sound pressure level metre during a pilot phantom session to be sure that the gradient noise is safe for participants in an experiment. Our rule is that we never run a pulse sequence louder than 110 dB on our participants (who, of course, have hearing protection), and it is easy to find settings that exceed that sound pressure level.

Solutions do exist to all of the above boundary conditions, and after finding the right combination of parameters, one typically does a pilot run to ensure that, with all of the above conditions satisfied, the temporal signal-to-noise ratio (tSNR) is acceptable. Every laboratory will have a different rule of thumb for acceptable tSNR. Our goal for an acquisition is a minimum of 20 (mean divided by the standard deviation of a short time series). Experience has taught us that only very patient participants with good intrinsic CNR will be able to provide analysable data if tSNR is below 10.

## 5. Characterizing blurring

In addition to characterizing tSNR for a new imaging protocol, it is valuable to characterize different sources of blurring; that is, degradations to spatial resolution in functional data. Since the earliest days of fMRI, a great deal of effort has been invested in understanding the spatial resolution of different techniques [10,102]. Resolution as it pertains to MRI can be broken down into three main categories: nominal resolution, image resolution and functional resolution. Nominal resolution refers to the voxel size specified by the field of view (on any given dimension) divided by the number of voxels in the image (on that dimension). Image resolution is determined by physics (i.e. acquisition choices) and is consistent for data from any object. Factors impacting image resolution may include sloped slice profiles [103], signal decay during acquisition (often referred to as T_2_* blurring [104]), displacement of signal by frequency perturbations [105] and artefacts introduced during reconstruction (e.g. zero-filling the *k*-space data after a partial Fourier (PF) acquisition (see [106,107] for informal discussions).

The functional resolution, on the other hand, refers to the spatial specificity of the blood flow or blood oxygenation signal that gives rise to functional contrast. Because the functional resolution is modulated by physiological sources (e.g. long-range neuronal connections, large vessels and/or pulse- and respiration-induced motion of the GM), it must be measured from *in vivo* data. Generally, blood flow- and blood volume-weighted methods that are sensitive to arterial regulation and capillary dilation have the potential for highest spatial specificity in the parenchyma (i.e. GM between pial and WM surfaces). T2-weighted approaches also have the potential for sub-millimetre spatial specificity because they are dominated by signals from post-capillary venuoles and the smaller branches of penetrating intracortical venuoles. On the other hand, T2*-weighted approaches have the greatest risk of contamination by signals from large veins that pool signal over several millimetres of cortex. These effects are separate from other physiological sources of blurring such as subject motion and respiration artefacts [108], and blurring introduced during pre-processing of data, which results from the details of the algorithms used for motion- and distortion-compensation. As a general rule, blurring increases SNR, so these two factors—the desire for high SNR and the desire for low blurring—conflict.

It is generally understood that functional resolution cannot be better than image resolution, and the image resolution will not be as good as the nominal resolution of the image. It is, however, difficult to separate the different sources of blurring and define objective metrics for quantifying blurring. For defining functional resolution, several studies have borrowed the idea of ‘point-spread function’ (PSF) from linear systems analysis and used visual stimuli to create regular patterns [109,110] or presumably sharp boundaries of neuronal response in the GM [111]. The sharpness of the corresponding fMRI response gives an indication of the functional blurring. These studies generally produce answers in agreement with our knowledge of vascular structure: T2*-weighted imaging methods have slightly more blurring (i.e. lower functional resolution) than T2-weighted imaging. The challenge of interpreting these data is that the underlying neural PSF is not defined, and because the dendritic arbours of pyramidal cells can span as much as 1 mm, it is likely that neuronal activity itself has a PSF approaching 1 mm. Furthermore, because of the heterogeneity of the vascular structure, not all voxels in a given region will have the same functional blurring [112].

For characterizing imaging resolution, one approach is to image a precisely machined grid phantom and compare the imaged size of features to their actual size (see [113]). Another approach we have been exploring for characterizing image resolution is quantifying the spatial autocorrelation function of the thermal noise (i.e. the noise attributable to system electronics rather than physiological fluctuations). To characterize imaging resolution in high-resolution images, the mean voxel intensity of the timeseries (i.e. the structure resulting from tissue contrast) is subtracted out, leaving only residual noise. Then, the correlation of voxel intensity at a single time point in an image volume is computed as it is shifted against itself one voxel at a time. As the shift gets larger, the correlation falls off. If the residual noise has no spatial structure (i.e. low thermal noise), the correlation will fall to zero after a single-voxel shift. Long-range spatial structure in the noise will reveal itself as a spatial autocorrelation function that persists after shifting the image against itself by several voxels.

The structure of the spatial autocorrelation of physiological noise has received much attention lately [114] and is best described by an exponential decay [115]. Thermal noise (which determines image resolution) typically has a Gaussian structure. In low-resolution images, with large voxel volumes, thermal noise is small compared to the signal strength; thus, the noise in the data is dominated by physiological sources [116,117] that limit the functional resolution. By contrast, the proportion of thermal noise to signal strength is larger in high-resolution images, and 98% or more of the autocorrelational structure of the combined noise can be described by a Gaussian kernel. The width of this Gaussian kernel provides a useful metric for estimating image resolution and tracking its degradation by zero-filling of *k*-space or motion- and distortion-compensation of data acquired *in vivo*.

**Figure 5.**
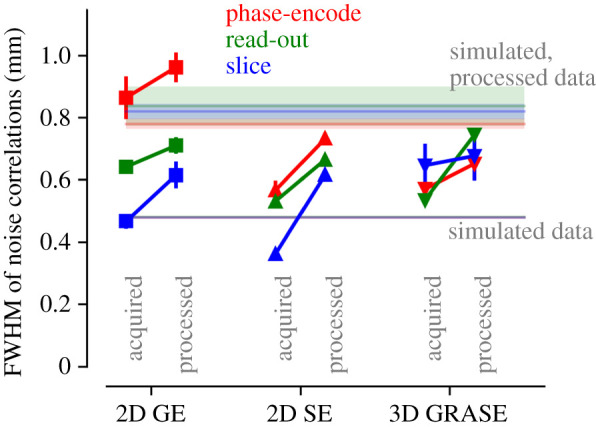
A cautionary tale about nominal and true resolution in isotropic 0.8 mm fMRI data acquisition. The horizontal line at 0.48 indicates the spatial noise correlation (full-width at half-maximum, FWHM) estimated by AFNI's *3dFWHMx* tool for synthetic, random (independently distributed) data in a matrix with 0.8 mm nominal resolution. FWHM estimates were derived from a Gaussian fit to spatial autocorrelations after voxel-wise detrending; 0.48 is a reasonable number for 0.8 mm voxels. The shaded regions at approximately 0.8 mm indicate the estimated FWHM after representative motion- and distortion-compensation processes were applied to the synthetic data (one-stop resampling, combining gradient nonlinearity correction, blip-up/blip-down unwarping and motion compensation with a wsinc5 algorithm applied by AFNI's *3dAllineate* algorithm during the final resampling step). Three features are notable: blurring in the slice direction (blue points) is initially absent in two-dimensional GE and SE EPI acquisitions but is, reasonably, introduced during motion- and distortion-compensation. Strong blurring in the phase-encode direction in the GE sequence (red squares) is caused by long read-out times and partial Fourier acquisition. Strong blurring in the slice direction in the three-dimensional GRASE acquisition could be eliminated by getting rid of partial Fourier under-sampling in the slice direction, at the cost of significant SNR reduction owing to increased echo train length. (Online version in colour.)

We illustrate the utility of this method for characterizing image blurring in the dataset shown in figure 5. The full-width at half-maximum (FWHM) of separate Gaussian kernels fit to the *x*-, *y*- and *z*- directions of the spatial autocorrelation of the image noise (the residuals after removing the mean signal from each voxel) in each dataset was used to estimate the image blurring after the acquisition and after pre-processing (see electronic supplementary material for details). Previous work [118] has characterized the image blurring in two-dimensional SE EPI and three-dimensional GRASE (each acquisition has a worst direction), and the data shown in figure 5 recapitulate that. On top of that, the blurring in the phase-encode direction for the particular two-dimensional GE EPI data shown in figure 5 was exacerbated by the use of PF acquisition. PF acquisition undersamples the data, taking advantage of the fact that one half of *k*-space can be inferred from the other half, because a real-world image is represented in frequency space by a complex-valued matrix with Hermitian symmetry. PF acquisition can, however, degrade image resolution because it introduces an asymmetry in *k*-space that equates to a convolution kernel in image space. To mitigate this problem, some systems estimate the missing data (using a variety of algorithms) when reconstructing PF images, but the downside to this approach is the possibility of creating image artefacts. The scanner used for this acquisition defaulted to doing nothing, leaving zeros in the matrix before doing the inverse Fourier transform to create the image. This multiplication by an asymmetric edge in the Fourier domain resulted in blurring in the phase-encode direction of the reconstructed image that nearly doubled the effective voxel size. A follow-up acquisition verified that full *k-*space acquisition, while detrimental to SNR, dramatically reduced blurring in the phase-encode direction. Thus, the two-dimensional GE EPI data in figure 5 are not representative of best practices, but rather a cautionary tale about attention to details when setting up a new protocol.

For sub-millimetre acquisitions in which SNR is hard to come by, yet it is of paramount importance to control image blurring, there are myriad other parameters that can and should be optimized. The best advice for understanding the details of how different scanners handle the details of image processing is not necessarily in peer-reviewed references but in conversations with physicists at conferences, white papers and blogs (e.g. Layer fMRI Blog, [114]). At the Cortical Depth-resolved fMRI Workshop—a part of the biannual Minnesota High Field Workshops—Dr. Huber (Faculty of Psychology and Neuroscience, Maastricht University) provided an excellent introduction to the tools that a person can access ‘under the hood’ on a scanner (https://shorturl.at/lyAE2). It makes sense that communication of the key tools for controlling image quality is not done by traditional, peer-reviewed publications because the solutions are unique to specific sites and applications. For this reason, the laminar fMRI community is investing significant effort in building an open information-sharing network to ensure that every investigator has access to relevant and timely information about acquisition and analysis techniques.

## 6. Closing remarks

A decade ago, UHF researchers had proven that cortical columns were accessible to fMRI experiments and were wondering whether the cortical vascular structure would permit depth-resolved functional MRI applications. In the past 5 years, we have seen proof that laminar fMRI profiles can be measured and do exhibit task-dependent and stimulus-dependent modulation that is consistent with known properties of the underlying neuronal populations [33]. Laminar fMRI is possible and, in fact, easier to verify than studies seeking to identify cortical columns because the GM and WM boundaries provide good landmarks for verifying the accuracy of laminar analyses. There have, however, also been many convincing demonstrations of the presence of confounding factors in both data acquisition and analysis.

The confounding factor that is the most important but also the most difficult to demonstrate is the potential for mis-registration between functional data and the anatomical boundaries that delineate the GM depth. It is clear that segmentation of GM on images that have the same distortion as the functional data is the best approach for addressing this problem, but that is not a readily achievable goal. The current challenge in the field is to standardize acquisition techniques, e.g. T_1_-weighted EPI to provide distortion-matched images that can be segmented, as well as analysis techniques (i.e. segmentation algorithms optimized for the contrast in T_1_-weighted EPI) to enable a routine solution to the problem of accurately defining GM boundaries in functional data.

If CNR and coverage were no object, all mesoscopic applications would use T_2_-, CBF- or CBV-weighted acquisition approaches that have been amply demonstrated to offer improved spatial specificity and truly beautiful images. As an extra advantage, VASO or CBF experiments that control for BOLD contamination by alternating between T_2_*- and T_1_-weighted acquisitions generate excellent contrast between GM and WM that can be used to address the registration problems discussed above. However, the limitations to coverage and CNR remain problematic, even with the advantages offered by 7 T field strength. The development of scanners that operate at even higher field (e.g. the 10.5 T system recently installed at our institution) can address some of the CNR problems, but not the problem of coverage.

While fMRI acquisition techniques other than GE EPI continue to catch up in terms of coverage (temporal resolution) and CNR, good progress can be made with T_2_*-weighted techniques as long as the field (investigators, reviewers and readers) remains mindful of the spatial confounds of macrovasculature. None of the techniques for removing these confounds—which displace signal in both radial and tangential directions, with respect to the cortical surface—is perfect. Deconvolution to remove vertical signal pooling requires modelling assumptions and high CNR; masking and GLM-based approaches can remove some but not all of the spatially non-selective signal.

The UHF laminar imaging field is in the fortunate position of having developed and communicated good tools for quantifying and addressing the known challenges of laminar fMRI. Continued open and informal communication of best practices is crucial to continued progress of the field. At the moment, the community is using a Slack team to facilitate communication between a network of users and hosting regular virtual workshops for informal discussion of current problems. The Slack sign-up link is posted at layerfmri.com, where virtual workshop announcements are also made. All readers are encouraged to monitor these sites to stay abreast of developments and participate in discussions.

## Supplementary Material

Details of scanning parameters for each data acquisition type.

## Data accessibility

The data presented in this work are openly available from OpenNeuro at https://openneuro.org/datasets/ds002684.
