# Supplementary material for: Forging a path to mesoscopic imaging success with ultra-high field functional magnetic resonance imaging
Source: Philos Trans R Soc Lond B Biol Sci. 2020 Nov 16;376(1815):20200040. doi: 10.1098/rstb.2020.0040 (PMC7741029; doi:10.1098/rstb.2020.0040)
Supplement: Details of scanning parameters for each data acquisition type. [file rstb20200040supp1.pdf]

Supplementary Material

Scanning Parameters

The comparative data were collected from four healthy adult participants (one male) who all gave informed written consent. The experiments presented in this work were approved by the University of Minnesota Institutional Review Board (1606M88867).

Functional MRI data were collected at the University of Minnesota's Center for Magnetic Resonance Research on a Siemens 7 Tesla scanner equipped with a SC-72 body gradient set (70 mT/m; 200 T/m/s) using 0.8 mm isotropic resolution. We used a custom 4-ch transmit, 32-ch receive head coil. The relative phases of the coil were constant for all participants and achieved a B_1 solution that provided adequate power throughout early visual regions. Functional volumes with reversed phase-encoding (PE) polarity (30 TRs, L/R PE) and anatomical scans (1 mm isotropic T_1-weighted MP-RAGE) were acquired in each session. Anatomical volumes (0.8 mm isotropic T_1-weighted MP-RAGE) were acquired for each participant in separate scanning sessions on a 3 Tesla system in the same facility.

Participants viewed an adapted population receptive field (pRF) mapping paradigm. They fixated on a central point during the presentation of bars moving across the visual field featuring highly-salient stimuli (e.g. faces, body parts, objects, or animals). During each scan, participants viewed 16 bar sweeps; each sweep lasted 16 s, with 4 s rest between, for a total scan duration of 324 s. The 2D GE EPI and 3D GRASE data were acquired with a 2 s TR and there were 4 pRF scans in the scanning session; the 2D SE data were acquired with a 2 s TR (although one scan had a 3 s TR) and there were 6 pRF scans in the scanning session, so all results show analysis of 648 TRs.

2D Gradient Echo

Gradient Echo (GE) data were acquired with a parallel imaging acceleration factor of 3 (6/8 Partial Fourier; echo-spacing = 1.0 ms; multiband = 1; TR = 2000 ms, TE = 23.4 ms, 36 coronal slices, right/left (R/L) PE direction).

The GE scanning session also included two different types of block-design visual stimuli, a "movie" scan, in which participants watched 12-sec clips of an animated movie interleaved with 12-sec of rest (mean gray screen), and a "checkerboard" scan, during which participants viewed a 4-Hz flickering checkerboard for 12-sec interleaved with 12-sec rest (mean gray screen). Visual stimuli were delivered to only one eye at a time. Both types of scans started and ended with left eye visual stimulus blocks and contained 6 blocks of stimulus to the left eye and 5 blocks of stimulus to the right eye.

2D Spin Echo

Spin Echo (SE) data were acquired with a parallel imaging acceleration factor of 2 (5/8 Partial Fourier; echo-spacing = 1.0 ms; multiband = 2; TR = 2-3000 ms, TE = 32 ms, 42 coronal slices, R/L PE direction).

3D GRASE

3D GRASE data were acquired with a parallel imaging acceleration factor of 2 (6/8 Partial Fourier; echo-spacing = 1.0 ms; TR = 2000 ms, TE = 36.3 ms, 12 coronal slices, head/foot PE direction).

MP2RAGE

MP2RAGE data (0.65 mm isotropic) were acquired with a parallel imaging acceleration factor of 2 (TR = 5000 ms, TE = 2.51 ms, R/L PE direction).
